# Supplementary material for: WordCommentsAnalyzer: A windows software tool for qualitative research
Source: F1000Res. 2018 Sep 4;7:536. Originally published 2018 May 3. [Version 2] doi: 10.12688/f1000research.14819.2 (PMC6137411; doi:10.12688/f1000research.14819.2)
Supplement: Supplementary file 3 [file f1000research-7-17614-s0002.tgz › b2a79496-4ada-47c4-9682-a99ce867836d.zip!/Eight Iranian Health Journals/Archives of Rehabilitation_23.docx]

© Archives of Rehabilitation. This work is licensed under CC BY-NC 4.0 (https://creativecommons.org/licenses/by-nc/4.0/).

Volume & Issue: دوره 14 - شماره 1

Title: بررسی ویژگی‌های شخصیتی در افراد معتاد و عادی با توجه به جنسیت

http://rehabilitationj.uswr.ac.ir/article-1-607-fa.html

هدف: هدف این پژوهش بررسی ویژگی‌های شخصیتی افراد معتاد و عادی با توجه به جنسیت بود.  روش بررسی: این مطالعه تحلیلی، به روش پس-رویدادی بر روی ۹۰ نفر با دامنه سنی۴۰-۲۰ سال (میانگین سن=۲۸/۴۱ و انحراف معیار=۵/۳۸) از مراجعین به مراکز بهزیستی و مراکز درمان سرپایی در بخش‌های مختلف شهر تبریز و مرند در سال ۱۳۸۸ که به طور تصادفی با روش نمونه‌برداری در دسترس انتخاب شده بودند انجام شد. گروه مورد بر مبنای ملاک‌های تشخیصی DSM-IV-TR به سوء مصرف یا وابستگی به مواد آمفتامین مبتلا بودند. گروه دیگر، ۶۰ نفر مرد، ۳۰ نفر زن (میانگین سن=۲۷/۷۱ و انحراف معیار=۴/۴۱) از افراد عادی جامعه که اعتیاد نداشته از بین اقوام، همسایگان و دوستان انتخاب شدند. مجموع نمونه شامل ۱۸۰ نفر بود. برای جمع‌آوری داده‌ها از آزمون شخصیت پنج عاملی نئو استفاده شد. برای تحلیل آماری از آزمون تحلیل واریانس چند متغیره و آزمون پیگیری (LSD) استفاده شد. یافته‌ها: نتایج نشان داد که تفاوت معنی‌داری بین گروه معتاد و عادی در ویژگی‌های شخصیتی وجود دارد. یافته‌ها حاکی از بالا بودن نمرات معتادان در عامل نوروزگرایی (۰/۰۰۱>P)، پایین بودن نمرات معتادان در عوامل‌های گشودگی در برابر تجربه (۰/۰۰۱>P)، توافق‌پذیری (۰/۰۰۱>P) و وجدان‌گرایی (۰/۰۰۱>P) بود. ولی تفاوت معنی‌داری در عامل برون‌گرایی (۰/۰۵>P)، توافق‌پذیری (۰/۰۵>P) و با وجدان بودن (۰/۰۰۱>P) بیشتر از مردان است. نمرات مردان نیز در عامل گشودگی در برابر تجربه (۰/۰۰۱>P) بیشتر از زنان بود. نتیجه‌گیری: نمره معتادان در پیوستار عوامل شخصیتی بیشتر به سوی منفی (شامل نورزگرایی، توافق‌پذیری، گشودگی در برابر تجربه و باوجدان بودن پایین) گرایش دارد. بنابراین، پیشنهاد می‌شود که در درمان معتادان یک ارزیابی گسترده از آنها به عمل آید و ویژگی‌های شخصیتی رشد نایافته در افراد معتاد شناسایی شده و سپس در کنار درمان دارویی به درمان این ویژگی‌ها نیز اقدام شود.

Title: بررسی و مقایسه مهارت‌های خواندن دانش‌آموزان شنوا و کم‌شنوای شدید پایه پنجم ابتدایی شهر تهران

http://rehabilitationj.uswr.ac.ir/article-1-677-fa.html

هدف: از آن‌جا که زبان نوشتاری مبتنی بر زبان گفتاری می‌باشد، احتمال می‌رود آسیب شنیداری باعث ایجاد نقایصی در مهارت‌های خواندن شود. هدف از این پژوهش بررسی نقایص خواندن کودکان کم‌شنوا و مقایسه مهارت‌های خواندن دانش‌آموزان کم‌شنوای شدید و شنوا در پایه پنجم ابتدایی می‌باشد. روش بررسی: در این مطالعه مقطعی مقایسه‌ای، ۱۶ کودک کم‌شنوا که در پایه پنجم مدارس باغچه‌بان تحصیل می‌کردند، با توجه به معیارهای ورود انتخاب و با ۱۶ کودک شنوا که براساس پایه تحصیلی با گروه نمونه همتاسازی شده بودند، مورد مقایسه قرار گرفتند. به منظور گردآوری داده‌ها از آزمون خواندن در دانش‌آموزان دبستانی و جهت تجزیه و تحلیل آنها از آزمونهای آماری کولموگروف-اسمیرنوف، تی مستقل و یومان ویتنی استفاده شد. یافته‌ها: یافته‌های پژوهش نشان داد که کودکان کم‌شنوا در مهارت‌های سرعت نامیدن (۰/۳۸۵=P)، حافظه شنیداری-کلامی‌صدا (معکوس) (۰/۳۴۵=P)، حافظه بینایی-کلامی‌تصویر (۱=P)، حذف واجی (۰/۸۱۷=P) و صحت خواندن ناکلمه (۰/۶۳۳=P) عملکرد مشابهی با گروه کنترل داشتند، ولی در مهارت‌های دیگر عملکرد ضعیف‌تری را نشان دادند.  نتیجه‌گیری: با توجه به نتایج تحقیق می‌توان اذعان داشت که پردازش شنیداری در همه مهارت‌های پیش نیاز و تکمیلی خواندن نقش کلیدی دارد و کودکان کم‌شنوا در تکالیف مبتنی بر پردازش‌های شنیداری و زبانی عملکردی ضعیف‌تر و در تکالیف مبتنی بر پردازش بینایی عملکردی مشابه با کودکان شنوا دارند.

Title: تحلیل رابطه بین اختلالات یادگیری و اختلالات زبان در دانش‌آموزان مقطع ابتدایی

http://rehabilitationj.uswr.ac.ir/article-1-731-fa.html

هدف: هدف از پژوهش حاضر، بررسی رابطه ی بین اختلالات یادگیری و اختلالات زبان در دانش آموزان مقطع ابتدایی بود. روش بررسی: روش این پژوهش توصیفی-تحلیلی از نوع همبستگی است. جامعه آماری این پژوهش کلیه دانش آموزان دچار اختلال یادگیری در مقطع ابتدایی شهرستان میبد بودند. نمونه مورد نظر در این پژوهش ۵۰ نفر (۲۵ دختر و ۲۵ پسر) از دانش آموزان دچار اختلال یادگیری بودند که به روش نمونه‌گیری در دسترس از مرکز اختلالات یادگیری این شهرستان که تحت آموزش و درمان بودند انتخاب شدند و با توجه به هدف و فرضیه های مطرح شده مورد آزمون قرار گرفتند. فرض اصلی این پژوهش این بود که بین اختلالات یادگیری و اختلالات زبان رابطه وجود دارد. ابزارهای مورد استفاده در این پژوهش، پرسش‌نامه سنجش اختلالات یادگیری میکل‌باست و پرسش‌نامه سنجش اختلالات زبان محقق ساخته و آزمون هوش کلامی وکسلر کودکان بود که پایایی و روایی آنها در حد مطلوبی بوده است. برای تجزیه و تحلیل داده ها از آزمون تی برای دو گروه مستقل، ضریب همبستگی پیرسون و تحلیل رگرسیون چند متغیری استفاده شد.  یافته‌ها: نتایج پژوهش رابطه معناداری را بین نمرات اختلال زبان و نمرات اختلال یادگیری نشان داد (۰/۵۵۸=r ؛ ۰/۰۵>P). همچنین بین خرده مقیاس‌های اختلال یادگیری و اختلال زبان رابطه معنادار بود. این نتایج در بین دانش آموزان پسر و دختر مشابه بود. نتیجه‌گیری: بر اساس یافته های پژوهش می‌توان نتیجه گرفت که تأخیر در رشد زبان و عناصر آن، می تواند یادگیری کودک را تحت تأثیر قرار دهد زیرا این همبستگی نشان می دهد که عوامل مشترکی بین محتوای یادگیری و زبان وجود دارد که هر دو جنبه از رشد را تحت تأثیر قرار می‌دهد. در واقع، در کنار عوامل تأثیرگذار بر اختلال یادگیری همچون آسیب های مغزی و عصبی، تأخیر در پیش نیازهای یادگیری تحولی از جمله رشد زبان، عامل دخیل مهمی محسوب می شود و خانواده باید نسبت به تحول زبانی کودک هم در قسمت درک و هم در قسمت گفتار توجه و مراقبت ویژه ای داشته باشد.

Title: مقایسه توانایی کنترل وضعیتی کودکان مبتلا به اختلال خواندن و کودکان عادی

http://rehabilitationj.uswr.ac.ir/article-1-760-fa.html

هدف: در برخی بررسی‌های انجام شده بر روی کودکان مبتلا به اختلال خواندن روابط معناداری بین توانایی کنترل وضعیتی و اختلال خواندن گزارش شده است. در این بررسی با کنترل عوامل مداخله کننده، این ارتباط با مقایسه کنترل وضعیتی و ویژگی‌های تعادلی بین کودکان عادی و کودکان مبتلا به اختلال خواندن بررسی شد. روش بررسی: مطالعه مورد-شاهد حاضر بر روی ۱۹ کودک پسر مبتلا به اختلال خواندن با میانگین سنی ۱۳/۷۸±۱۱۲/۹۰ و ۱۹ کودک پسر عادی با میانگین سنی ۱۵/۶۲ ± ۱۱۸/۴۲ ماه صورت گرفته است. دو گروه از نظر سن، قد و وزن با هم یکسان می‌باشند. تکالیف وضعیتی شامل ایستادن ساکن با پاهای مجاور هم روی سطح سفت با چشم باز و بسته، روی اسفنج با چشم بسته و روی سطح سفت بهمراه اغتشاشات داخلی بوده است. مدت زمان انجام هر یک از تکالیف ۳۵ ثانیه بود و از دستگاه صفحه نیرو برای ارزیابی عملکرد وضعیتی استفاده شد. از تمامی‌نمونه‌ها، قسمت تعادل تست برونینکز اوزرتسکی نیز گرفته شد و همبستگی تست‌های عملکردی و تست‌های آزمایشگاهی مورد بررسی قرار گرفت. آزمون‌های آماری کولموگروف-المیرونوفت وتی مستقل و ضریب همبستگی پیرسون جهت تحلیل داده‌ها استفاده شد. یافته‌ها: نتایج نشان داد که سطح کل ایستادن روی سطح سفت با چشم باز بین دو گروه تفاوت معناداری دارد (۰/۰۵>P). هم‌چنین وابستگی به اغتشاشات داخلی در انحراف معیار سرعت در راستای داخلی-خارجی، قدامی-خلفی و هم‌چنین سطح کل تفاوت معناداری بین دو گروه کودکان مبتلا به اختلال خواندن و کودکان عادی دارد، ولی طول مسیر و متوسط سرعت در حالت‌های مختلف کنترل وضعیتی (فوم، سطح سفت، چشم باز و بسته) و وابستگی به بینایی در تمامی‌ پارامترهای طول مسیر، سرعت و سطح کل تفاوت معناداری بین دو گروه کودکان وجود نداشت. هم‌چنین ارتباط معناداری بین پارامترهای مرکز فشار با بخش تعادلی تست برونینکز اوزرتسکی در کودکان مبتلا به اختلال خواندن یافت نشد.  نتیجه‌گیری: با وجود تفاوت در برخی پارامترهای کنترل وضعیتی بین کودکان مبتلا به اختلال خواندن و عادی، ارتباط معناداری بین کنترل وضعیتی و اختلال خواندن یافت نشد.

Title: رابطه هوش‌هیجانی، کیفیت زندگی و ویژگی‌های شخصیتی با سلامت روان در افراد با معلولیت (ناتوانی) جسمی حرکتی

http://rehabilitationj.uswr.ac.ir/article-1-826-fa.html

هدف: پژوهش حاضر به منظور بررسی رابطه هوش‌هیجانی، کیفیت زندگی و ویژگی‌های شخصیتی با سلامت روان در افراد با معلولیت (ناتوانی) جسمی حرکتی انجام شد.   روش بررسی: در قالب یک طرح همبستگی، با استفاده از روش نمونه‌گیری تصادفی ساده از میان معلولین جسمی حرکتی مجتمع نیکوکاری آموزشی توان‌یابان مشهد تعداد ۶۰ نفر (۳۰ دختر و ۳۰ پسر) انتخاب شدند و پرسش‌نامه سلامت عمومی (GHQ)، پرسش‌نامه هوش‌هیجانی بار-آن، پرسش‌نامه ویژگی‌های شخصیت ( NEO – FFI ) و پرسش‌نامه کیفیت زندگی (WHOQOL–BREF) را تکمیل نمودند. تجزیه و تحلیل داده‌ها با استفاده از ضریب همبستگی پیرسون و تحلیل رگرسیون چندگانه انجام شد.   یافته‌ها: تحلیل داده‌ها نشان داد که ویژگی‌های شخصیتی و کیفیت زندگی با چهار بعد سلامت روان همبستگی مثبت و معناداری دارند ( 0/86=r؛ 0/87=r؛ 0/05>P)، در حالی که بین مؤلفه‌های هوش هیجانی با سلامت روان همبستگی معناداری وجود ندارد (0/03-=r؛ 0/05P) ولی بین مؤلفه‌های هوش هیجانی با ویژگی‌های شخصیت و کیفیت زندگی همبستگی منفی است و معنادار نمی‌باشد. (0/05)   نتیجه‌گیری: ویژگی‌های شخصیت و مؤلفه‌های آن و نیز کیفیت زندگی با مؤلفه‌های سلامت روان رابطه‌ای مستقیم داشته و قادر به پیش‌بینی آن می‌باشند.

Title: بررسی مقایسه‌ای تأثیر تمرینات ثباتی و کششی-تقویتی بر شاخص‌های تعادلی در افرادی با وضعیت جلو آمده سر

http://rehabilitationj.uswr.ac.ir/article-1-979-fa.html

هدف: اختلال تعادل یکی از اختلالات مشاهده شده در افرادی با وضعیت جلوآمده سر می‌باشد. هدف از این مطالعه مقایسه تأثیر تمرینات ثباتی و استرچی-تقویتی بر بهبود تعادل در این بیماران بود. روش بررسی: در این پژوهش تجربی و مداخله‌ای سی و سه زن مبتلا به وضعیت جلو آمده سر به طور تصادفی در یکی از سه گروه از تمرینات ثباتی، کششی-تقویتی و کنترل قرار گرفتند. میزان جلوآمدگی سر از طریق خط شاقول و زاویه کرانیو-ورتبرال اندازه‌گیری شد. آزمون‌های تعادلی در سه وضعیت مختلف ایستادن روی صفحه نیرو انجام شد. افراد پس از شش هفته از انجام تمرین‌درمانی و یک ماه دوره پیگیری، از طریق آزمون آنالیز واریانس در تکرار مشاهدات و آزمون‌ تی مستقل مورد ارزیابی قرار گرفتند. یافته‌ها: شاخص تعادلی در دو گروه کنترل و کششی-تقویتی، تغییر معنی‌داری نشان نداد، در حالی که در گروه ثباتی، انحراف معیار جابه‌جایی مرکز فشار در جهت قدامی-خلفی (۰/۰۲=P) و انحراف معیار سرعت مرکز فشار در جهت داخلی- خارجی (۰/۰۴=P) در وضعیت دوپا-چشم باز-سطح‌نرم و انحراف معیار سرعت مرکز فشار در جهت قدامی-خلفی (۰/۰۱=P) در وضعیت یک پا-چشم بسته-سطح سخت کاهش قابل توجهی نشان داد. هیچ تغییری در شاخص‌های تعادل در وضعیتی که فرد روی دو پا-چشم باز-سطح سخت ایستاده بود، اتفاق نیافتاد. نتیجه‌گیری: در وضعیت‌هایی از ایستادن که اطلاعات بینایی و حس عمقی از ناحیه کف پا به چالش کشیده می‌شود، تمرینات ثباتی بیش از تمرینات کششی-تقویتی سبب بهبود تعادل می‌گردد.

Title: بررسی تأثیر استفاده از کاشت حلزون و برنامه توانبخشی شنوایی در پیشرفت مهارت‌های شنوایی کودکان استفاده کننده از کاشت حلزون

http://rehabilitationj.uswr.ac.ir/article-1-991-fa.html

هدف: مطالعه حاضر با هدف تعیین تأثیر استفاده از کاشت حلزون و برنامه توانبخشی شنوایی در پیشرفت مهارت‌های شنوایی کودکان استفاده کننده از کاشت حلزون انجام گرفت. روش‌بررسی: مطالعه طولی تحلیلی حاضر روی ۱۵ کودک دچار کم‌شنوایی شدید تا عمیق با میانگین سنی ۴۵/۲۷ ماه صورت گرفت که در مرکز کاشت حلزون بیمارستان امیراعلم از مرداد تا دی‌ماه ۱۳۸۹ تحت عمل کاشت حلزون قرار گرفتند. افراد به صورت غیر احتمالی تدریجی انتخاب شدند. ارزیابی مهارت شنوایی با استفاده از آزمون سنجش رشد نیوشا به صورت پرسش از والدین کودکان کم‌شنوا پیش از جراحی و پس از آن به مدت ۸ ماه در فواصل ۲ ماهه صورت گرفت. پس از تعیین سن رشدی کودک، سرعت رشد کودک در زمان پیش‌آزمون، شاخص کارایی مداخله و شاخص کارایی برنامه توانبخشی محاسبه شد. برای تحلیل داده‌ها از آزمون آماری آنوای یک سویه و تحلیل پوست هوک استفاده شد.  یافته‌ها: افزایش معناداری در سرعت رشد کودک طی انجام برنامه توانبخشی مشاهده شد (۰/۰۰۱>P). بین شاخص کارایی مداخله طی ۴ بار ارزیابی دوره‌ای، تفاوت معناداری وجود داشت (۰/۰۰۱>P). هم‌چنین، اختلاف معناداری بین شاخص کارایی برنامه توانبخشی شنوایی طی ۴ بار ارزیابی دوره‌ای مشاهده شد (۰/۰۰۱>P). نتیجه‌گیری: انجام برنامه توانبخشی شنوایی پس از کاشت حلزون به تسریع رشد مهارت‌های شنوایی کودکان کم‌شنوا و نزدیک شدن به سطوح شنوایی متناسب با سن منجر می‌شود.

Title: اثربخشی آموزش کارکردهای اجرایی (بازداری پاسخ) بر میزان تکانشگری کودکان پیش‌دبستانی دارای اختلال هماهنگی رشد: پژوهش موردی

http://rehabilitationj.uswr.ac.ir/article-1-1049-fa.html

هدف: بررسی تأثیر آموزش کارکردهای اجرایی (بازداری پاسخ) بر میزان تکانشگری کودکان پیش‌دبستانی دارای اختلال هماهنگی رشد شهر اصفهان بود.  روش بررسی: جامعه آماری پژوهش موردی حاضر شامل کلیه کودکان پیش‌دبستانی (۶-۵ ساله) دارای اختلال هماهنگی رشد بود که در مراکز پیش‌دبستانی و آمادگی وابسته به آموزش و پرورش شهر اصفهان ثبت نام شده بودند. روش نمونه‌گیری هدفمند بود. آزمودنی‌ها ۳ کودک پیش‌دبستانی بودند که به تشخیص متخصص دارای علائم و نشانه‌های اختلال هماهنگی رشد بوده‌اند. ابزارها شامل آزمون عصب-روان‌شناختی نپسی، پرسش‌نامه کانرز والدین، مقیاس هوشی وکسلر کودکان، آزمون توانایی-حرکتی پایه و مصاحبه بالینی بود. در این پژوهش از روش موردی با طرح MBD استفاده شد. یافته‌ها: یافته‌ها نشان داد که طی تحلیل دیداری نمودار داده‌ها براساس شاخص‌های آمار توصیفی و تحلیل دیداری، مداخله مورد نظر در مورد هر سه آزمودنی اثر بخش بوده است (به ترتیب با %PND ۸۰% ،۷۰ و ٪۷۰ برای آزمودنی شماره یک، دو و سه). نتیجه‌گیری: نتایج پژوهش حاضر نشان داد که با آموزش کارکرد اجرایی بازداری پاسخ، میزان تکانشگری کاهش پیدا می‌کند.

Title: بررسی تأثیر پیچیدگی نحوی ساختار گروه اسمی ‌و فعلی بر وقوع لکنت در کودکان لکنتی پیش‌دبستانی ۶-۴ سال فارسی‌زبان

http://rehabilitationj.uswr.ac.ir/article-1-1128-fa.html

هدف: هدف پژوهش حاضر بررسی تأثیر پیچیدگی نحوی ساختار گروه اسمی ‌و گروه فعلی بر وقوع لکنت در کودکان پیش‌دبستانی فارسی زبان ۶-۴ سال است. روش بررسی: این پژوهش توصیفی-تحلیلی بر روی ۱۵ کودک لکنتی، شامل ۱۲ پسر و ۳ دختر، ۶-۴ ساله فارسی‌زبان تک زبانه که به کلینیک گفتاردرمانی جوادالائمه شهر مشهد مراجعه نمودند، انجام گرفت. روش نمونه‌گیری، نمونه‌گیری آسان ( در دسترس) بود. صدای این کودکان به وسیله یک ام‌.پی‌.تری ضبط گردید و یک نمونه گفتار خودانگیخته ۳۰ دقیقه‌ای از هر یک از کودکان لکنتی مورد نظر ذخیره شد. این کودکان، حدود ۶۰ پاره‌گفتار را در حین تعامل با گفتاردرمانگر، والد (پدر یا مادر) و یا پژوهشگر تولید کردند. نمونه گفتار تولید شده توسط هر یک از این ۱۵ کودک لکنتی روی کاغذ واج‌نگاری شد. سپس تحلیل داده‌های زبانی فقط به صورت گروهی و با روش آزمون‌تی زوجی صورت گرفت. یافته‌ها: تحلیل گروهی، تفاوت‌های مهمی‌ را بین پاره‌گفتارهای روان و ناروان از نظر پیچیدگی نحوی ساختار گروه اسمی‌ و فعلی نشان داد. به‌علاوه، یافته‌های پژوهش حاکی از آن است که در سطح گروه، در گروه اسمی‌ برحسب سه نقش آن در جمله به عنوان نهاد، مفعول مستقیم و مفعول حرف اضافه، فقط تعداد نهاد (۰/۰۰۱>P) و مفعول حرف اضافه (۰/۰۵۰=P) بطور معناداری با وقوع لکنت ارتباط دارند. در گروه فعلی، بین سه متغیر حضور فعل کمکی، فعل ربطی و پیشوند منفی‌ساز فقط حضور فعل کمکی با وقوع لکنت از ارتباط معناداری برخوردار است (۰/۰۱۰=P). نتیجه‌گیری: نتایج این پژوهش نشان می‌دهد که، در کودکان لکنتی فارسی زبان ۶-۴ سال پیش‌دبستانی، بین متغیر پیچیدگی نحوی برحسب ساختار گروه اسمی‌ و فعلی و وقوع لکنت ارتباط معناداری وجود دارد.

Title: مقایسه توزیع فشار کف‌پایی و نیروی عکس‌العمل عمودی زمین در اندام غالب و غیرغالب افراد سالم با استفاده از تکنیک آنالیز اجزای اصلی

http://rehabilitationj.uswr.ac.ir/article-1-1168-fa.html

هدف: هدف این مقاله، مقایسه توزیع فشار کف‌پایی و نیروی عکس‌العمل عمودی زمین در اندام‌غالب و غیرغالب افراد سالم با استفاده از تکنیک آنالیز اجزای اصلی (PCA) بود.  روش بررسی: در این تحقیق تحلیلی و مقایسه‌ای ۲۰ مرد و زن بالغ سالم از بین نمونه‌های در دسترس به صورت ساده انتخاب شدند. توزیع فشار کف‌پایی و نیروی عکس‌العمل عمودی زمین با استفاده از سیستم پدار مورد اندازه‌گیری قرار گرفت. اطلاعات به دست آمده با استفاده از آزمون‌های تی زوج، آنالیز واریانس یک سویه (ANOVA)، ضریب همبستگی پیرسون و PCA مورد تجزیه و تحلیل آماری قرار گرفت. یافت&zwjه‌ها: تفاوت معناداری در الگوی توزیع فشار مردان و زنان و هم‌چنین پای غالب و غیر‌غالب وجود نداشت. برای سمت غالب فشار و نیروی مربوط به پاشنه، انگشتان، انگشت شست و متاتارس اول و در سمت غیرغالب فشار و نیروی مربوط به ناحیه خارج وسط پا، ناحیه خارج جلوی پا، متاتارس اول و پاشنه نقاط مهم شناخته شد. نتیجه‌گیری: به نظر می‌رسد سمت غالب مسئولیت پیشروی و پذیرش وزن و سمت غیرغالب مسئولیت کنترل تعادل را بر عهده داشته باشد. بنابراین در هنگام بررسی بیومکانیکی عملکرد پنجه، غالب یا غیرغالب بودن آن باید مدنظر قرار گیرد.

Title: بررسی پاسخ‌های شنیداری برانگیخته ساقه مغز در کودکان مبتلا به سندرم داون

http://rehabilitationj.uswr.ac.ir/article-1-1116-fa.html

هدف: هدف از این بررسی، مقایسه پاسخ‌های شنیداری برانگیخته ساقه مغز کودکان هنجار و نیز مبتلا به سندرم داون می‌باشد. روش بررسی: این مطالعه مقطعی و مقایسه‌ای در مرکز توانبخشی اخوان دانشگاه علوم بهزیستی و توانبخشی تهران و بیمارستان تخصصی و فوق تخصصی کودکان امیرکلا بابل در محدوده سال‌‌های ۱۳۸۸ تا ۱۳۹۱ انجام شد. ۴۵ کودک ۳ تا ۶ ساله مبتلا به سندرم داون و ۴۵ کودک هنجار از نمونه در دسترس و از میان پسرها انتخاب شدند. پس از تاریخچه‌گیری، اتوسکوپی و ارزیابی پایه شنوایی، آزمون پاسخ‌های شنیداری برانگیخته ساقه مغز (ABR) انجام شد. پارامتر‌های مورد مطالعه در ABR عبارتند از: نهفتگی مطلق امواج I، III و V، نهفتگی بین امواج و نسبت دامنه موج V/I. برای تجزیه و تحلیل داده‌ها از آزمون پارامتری t مستقل استفاده شد. یافته‌ها: نهفتگی مطلق امواج ABR و نهفتگی بین امواج I-III و I-V؛ (۰/۰۰۱>P)، و نهفتگی بین امواج (III-V (P=۰/۰۰۳ در گروه مبتلا به سندرم داون به شکل معناداری (۰/۰۰۱=P) از گروه هنجار کوتاه‌‌تر بود. نسبت دامنه V/I در گروه مبتلا به سندرم داون به شکل معناداری کوچک‌‌‌تر از گروه هنجار (۰/۰۰۱>P) و آستانه موج V در گروه مبتلا به سندرم داون به شکل معناداری از گروه هنجار بالا‌‌تر بود (۰/۰۰۱>P). نتیجه‌گیری: رشد دستگاه شنوایی محیطی در کودکان مبتلا به سندرم داون تأخیر داشته و عملکرد ساقه مغز غیرهنجار است. تشخیص به‌موقع اختلالات شنوایی و مداخله زودهنگام در این کودکان ضروری است زیرا این اختلالات با توانایی‌‌های ارتباطی کودک مداخله می کند.

Title: خانواده، اصلی ترین مأمن بیماران افسرده

http://rehabilitationj.uswr.ac.ir/article-1-1111-fa.html

هدف: افسردگی شایع‌ترین اختلال خلقی است. امروزه قسمت اعظم مسئولیت مراقبت از بیماران روانی برعهده خانواده‌ها می‌باشد. خانواده‌ها برای مراقبت بهینه و اصولی از عضو افسرده، نیاز به حمایت سیستم‌های اجتماعی و درمانی دارند. مطالعه حاضر به منظور تبیین تجارب خانواده‌های بیماران افسرده از عملکرد سیستم‌های حمایتی انجام شده است. روش بررسی: این مطالعه با روش کیفی و رویکرد تحلیل محتوای کیفی از نوع قراردادی انجام گرفته است. ۲۴ نفر از اعضای خانواده بیماران افسرده که به درمانگاه روانپزشکی رازی مراجعه کرده بودند، به روش مبتنی بر هدف برای مصاحبه انتخاب شدند. مصاحبه‌ها به روش ساختار نیافته و عمیق انجام شد. کلیه مصاحبه‌ها پیاده و با آنالیز مداوم و مقایسه‌ای مورد تجزیه و تحلیل قرار گرفتند. نرم‌افزار ۲۰۰۷MAXQDA برای مدیریت داده‌ها مورد استفاده قرار گرفت. یافته‌ها: طبق تجارب خانواده‌ها، منابع حمایتی-اجتماعی و درمانی، جامع و کافی نیستند. نحوه عملکرد منابع حمایتی در چهار درون‌مایه استخراج شدند. «نگاه نامناسب جامعه»، «حمایت ناکافی»، «سیستم‌درمانی غیر مؤثر» و «فقر اقتصادی-شناختی خانواده» درون‌مایه‌های اصلی بودند. نتیجه‌گیری: خانواده‌ها در فرآیند مراقبت از عضو افسرده، احساس تنهایی می‌کنند. در فرایند مراقبت از بیمار افسرده لازم است که خانواده‌ها از منابع حمایتی منسجم و در دسترس برخوردار باشند و تیم‌درمانی برنامه‌های پیگیری درمان را بطور مستمر و پویا انجام داده و با ارائه خدمات مراقبتی و مشاوره‌ای در منزل به خانواده‌ها کمک کنند.

Title: تأثیر درمان‌های دستی بر روی اسکولیوز با علت نامعلوم در یک خانم ۲۸ ساله-گزارش موردی

http://rehabilitationj.uswr.ac.ir/article-1-929-fa.html

اسکولیوز با علت نامعلوم شایع‌ترین نوع اسکولیوز می‌باشد. علایم و نشانه‌های این بیماری بر کیفیت زندگی و عملکرد بیماران تأثیر بسزایی دارد. تکنیک‌های دستی که توسط کایروپرکتورها و استئوپات‌ها انجام می‌شود، معمولاً در درمان این بیماران مورد استفاده قرار می‌گیرد. . این یک گزارش موردی از درمان اسکولیوز با علت نامعلوم به کمک درمان‌های دستی در یک بیمار ۲۸ ساله می‌باشد که به مدت ۴ سال از کمردرد رنج می‌برده است. به کمک رادیوگرافی مشخص شد که‌این بیمار مبتلا به اسکولیوز با زاویه‌ انحراف ۳۲ درجه است. بیمار به مدت ۱۶ هفته و دو جلسه در هر هفته، تحت درمان با تکنیک‌های آزادسازی میوفاسیا، موبیلیزاسیون و مانیپولاسیون قرار گرفت. پس از ۸ هفته درمان میزان زاویه‌ انحراف به‌اندازه ۱۵٫۶۲٪ کاهش یافت و پس از ۱۶هفته درمان میزان این زاویه به‌اندازه %۲۱/۸۷ کاهش یافت. هم‌چنین درد بیمار به میزان ۵۰٪ کم شد، می‌توان نتیجه گرفت که درمان‌های دستی باعث بهبود اسکولیوز با علت نامعلوم در این بیمار گردید.
